# Efficacy of post-inpatient aftercare treatments for anorexia nervosa: a systematic review of randomized controlled trials

**DOI:** 10.1186/s40337-021-00487-5

**Published:** 2021-10-15

**Authors:** Katrin E. Giel, Simone C. Behrens, Kathrin Schag, Peter Martus, Stephan Herpertz, Tobias Hofmann, Eva-Maria Skoda, Ulrich Voderholzer, Jörn von Wietersheim, Beate Wild, Almut Zeeck, Ulrike Schmidt, Stephan Zipfel, Florian Junne

**Affiliations:** 1 Department of Psychosomatic Medicine and Psychotherapy, Medical University Hospital Tübingen, Eberhard Karls University, Osianderstr. 5, 72076 Tübingen, Germany; 2 Center of Excellence in Eating Disorders, Tübingen, Germany; 3 Institute for Clinical Epidemiology and Applied Biostatistics, Medical Faculty, Eberhard Karls University Tübingen, Tübingen, Germany; 4 Department of Psychosomatic Medicine and Psychotherapy, LWL-University Hospital Bochum, Ruhr University Bochum, Bochum, Germany; 5 Department of Psychosomatic Medicine, Center for Internal Medicine and Dermatology, Charité - Universitätsmedizin Berlin, Freie Universität Berlin and Humboldt-Universität Zu Berlin, Berlin, Germany; 6 Clinic for Psychosomatic Medicine and Psychotherapy, LVR University-Hospital Essen, University of Duisburg-Essen, Essen, Germany; 7 Schoen Clinic Roseneck, Prien am Chiemsee, Germany; 8 Department Psychiatry and Psychotherapy, University Hospital LMU Munich, Munich, Germany; 9 Department Psychiatry and Psychotherapy, University Hospital Freiburg, Freiburg, Germany; 10 Department of Psychosomatic Medicine and Psychotherapy, Ulm University Medical Center, Ulm, Germany; 11 Department of General Internal Medicine and Psychosomatics, University Hospital Heidelberg, Heidelberg, Germany; 12 Department of Psychosomatic Medicine und Psychotherapy, Center for Mental Health, Faculty of Medicine, University of Freiburg, Freiburg, Germany; 13 Section of Eating Disorders, Department of Psychological Medicine, Institute of Psychiatry, Psychology and Neuroscience, King’s College London, London, UK; 14 Department of Psychosomatic Medicine and Psychotherapy, University Hospital Magdeburg, Otto von Guericke University, Magdeburg, Germany

**Keywords:** Aftercare, Anorexia nervosa, Eating disorder, Efficacy, Randomized-controlled trials, Relapse, Treatment, Therapy

## Abstract

**Background:**

Early relapse after inpatient treatment is a serious problem in the management of anorexia nervosa (AN). Specialized aftercare interventions have the potential to bridge the gap between inpatient and outpatient care, to prevent relapse and to improve the long-term outcome for patients with AN.

**Methods:**

Following the guidelines of the PRISMA statement, we conducted a systematic review, synthesizing the evidence from randomized-controlled trials (RCTs) investigating the efficacy of post-inpatient aftercare treatments for AN.

**Results:**

Our search resulted in seven RCTs and three registered ongoing trials. Pharmacotherapy and low-threshold guided self-help have limited uptake and high dropout. Novel mobile guided self-help approaches seem promising due to high patient satisfaction, but their efficacy has yet to be investigated in larger trials. Cognitive-behavior psychotherapy may be beneficial in delaying relapse, but evidence is based on a single study.

**Conclusion:**

Only a limited number of RCTs investigating aftercare interventions for patients with AN is available. There is no clear evidence favoring any one specific approach for post-inpatient aftercare in adult patients with AN. The field faces many challenges which generally affect intervention research in AN. A specific issue is how to increase uptake of and reduce dropout from aftercare interventions. This calls for better tailoring of interventions to patient needs and the integration of patient perspectives into treatment. Intensified research and care efforts are needed to address the problem of recurrent relapse after intensive inpatient treatment for AN and to eventually improve prognosis for this eating disorder.

## Plain English summary

Patients with a severe form of anorexia nervosa (AN) are often treated as inpatients. Many of them benefit from this acute treatment. Unfortunately, a significant number of patients experience relapse after discharge. This problem could be addressed by specific treatments directly following inpatient therapy, so called aftercare interventions, which are tailored to patients’ needs in this treatment period. This review looks at studies which have investigated the efficacy of aftercare interventions for patients with AN directly after inpatient treatment. We included any studies which compared a novel aftercare intervention to a control treatment and where patients were randomly assigned to either of these treatments, as this procedure is considered to reduce bias. We found seven studies that investigated different aftercare intervention approaches, including medication, guided self-help and psychotherapy, and three ongoing studies. Based on the very limited evidence so far, no clear recommendations can be made favoring a specific approach for post-inpatient aftercare in adult patients with AN. The review shows that it should be a priority to increase uptake of aftercare interventions and to reduce dropout rates. This could be achieved by a better tailoring of interventions to patient needs and the integration of patient perspectives in intervention design. More studies are needed to find interventions which allow patients with AN to maintain treatment gains after intensive inpatient treatment.

## Introduction

Anorexia nervosa (AN) is a severe mental disorder which often has a long-lasting and fluctuating course [1]. Up to 70% of patients with a severe course do not overcome the eating disorder (ED) in the long-term, and around 5% of patients die in long-term observation periods, yielding the highest mortality rate among all mental disorders [2–4]. A specific challenge in AN treatment, affecting all treatment settings and stages, is treatment adherence, with treatment discontinuation rates as high as 30–40% [5, 6]. To a certain extent this might reflect the ambivalence many patients experience towards weight gain and recovery [1, 7].

International treatment guidelines for EDs concur in their recommendation that psychotherapy is the first- line treatment for patients with AN, preferably in outpatient settings [8, 9]. There is currently no empirical evidence for the superiority of one specific psychotherapy approach in the treatment of adults with AN [8, 10]. For severely affected patients it is recommended that they are treated in specialized inpatient or day-hospital settings [8, 9]. However, these intensive acute treatments are not thought to replace outpatient therapy, but rather to enable it. Treasure et al. [11], concluded that continuous care which is matched to the stage of the illness may improve treatment outcomes. In line with this idea, treatment guidelines and recent suggestions for optimizing care pathways highlight the need for attending to the transition between different treatment settings in AN care [8, 12]. Related to this, the importance of ensuring post-inpatient care to prevent relapse and re-hospitalization has also been highlighted [12–14].

Regarding the implementation of these recommendations, evidence shows that inpatient treatment for AN is effective for many severely ill patients as they achieve weight gain and improvements in ED symptoms at least in the short-term [6, 10, 15]. Unfortunately, a considerable proportion of patients with AN experience deterioration or relapse in the first months after termination of inpatient treatment [16, 17]. This contributes to the unfavorable prognosis of AN [18] and also indicates that the post-discharge stage and transition from acute inpatient treatment to less intensive settings is a critical phase of transition which is currently not sufficiently addressed and managed in AN care. Accordingly, recent reviews and treatment guidelines point out that discharge from inpatient treatment often results in discontinuity of care and so far, empirical evidence about how to link intensive treatments with outpatient psychotherapy is scarce [1, 10, 13, 14, 17].

In addition to ensuring continuity of care, a post-inpatient intervention should ideally entail an approach that is tailored to the patient’s illness stage and needs, i.e. provide a specialized maintenance or aftercare treatment [13, 16]. Typical components of interventions focusing on maintenance and relapse prevention comprise an assessment of the stage of illness and symptom profile, recovery and relapse history, psychoeducation on relapse processes, strengthening treatment motivation, identification and monitoring of high-risk situations for relapse, identification of coping strategies to prevent and manage relapse as well as strengthening of individual resources [19]. These key components of maintenance treatment have been most widely implemented and researched in the context of addictive behaviors [19]. However, they might also be effective in relation to the maintenance of behaviour change in other mental disorders, but with disorder-specific adaptations, i.e. to target maintenance mechanisms which have been identified for the specific core symptoms of the disorder in question. This could generally be achieved by a range of approaches, including pharmacological interventions.

As mentioned above, guidelines suggest that there is currently not enough evidence regarding which types of interventions can reduce the risk of relapse after successful inpatient treatment of AN [13]. Two further aspects might add to the difficulties of developing and investigating aftercare interventions in AN: Many adult patients with AN do not leave specialized inpatient or day-hospital treatment in a weight-recovered or fully remitted state, but are still underweight and at least partially fulfill diagnostic criteria for AN or another ED at the point of discharge [6]. This situation poses specific challenges for the treatment goals and components of effective aftercare, in terms of balancing a focus on maintaining gains achieved during inpatient care versus initiating further improvement and weight gain. When it comes to evaluating outcomes and efficacy of any aftercare interventions, a further challenge is the lack of any consensus definitions of relapse or recovery in AN [17, 20].

To summarize, early relapse after inpatient treatment is a common and serious problem in the management of adult patients with AN [16, 17] which contributes to the often lengthy course of this ED [18]. There are challenges in the development and evaluation of specialized, targeted aftercare interventions for AN. In order to summarize existing knowledge on which treatments might help patients with AN to maintain benefits after inpatient or day-hospital treatment, we conducted a systematic review synthesizing current evidence from randomized controlled treatment trials (RCTs). We decided to include only studies with a RCT design as this is considered the gold standard for efficacy research. The present review complements recent reviews which focused on identifying established core strategies of existing and emerging treatments for acute AN [21, 22], but did not focus on the transition between different treatment settings.

The aims of this systematic review are (1) to identify aftercare treatments following inpatient or day-hospital treatment for patients with AN, (2) to analyze their efficacy and (3) to explore how recovery processes and outcomes are operationalized in the different studies.

## Method

A systematic literature research was performed following the PRISMA guidelines [23].

### Search strategy

PubMed, PsychInfo, and Web of Science were searched for combinations including “eating disorders or anorexia nervosa”, “relapse or maintenance” and “therapy, intervention, treatment or program”. We did not use any limits in our search. We further searched the International Clinical Trials Registry Platform (ICTRP), German Clinical Trials Register (DRKS), ISRCTN registry, Clinicaltrials.gov and the Cochrane Controlled Trial Register for completed and ongoing trials. Search results were included into the analyses until 31.12.2020.

### Eligibility criteria

After duplicates were removed, all records were screened and two raters (KEG and SCB) independently evaluated eligibility of the remaining studies. Disagreement was resolved through discussion and by integrating a further rater. Eligibility criteria were based on the PICOS taxonomy (participants, intervention, comparator, outcome, study design) according to the PRISMA statement [23]. Studies were considered eligible for the review if they met the following criteria:

(1) *Participants*: Examination of adult patients who had been diagnosed with AN or atypical AN at admission to acute treatment (inpatient / day-hospital treatment); (2) *Intervention*: treatment delivered after inpatient or day-hospital treatment and aiming at maintenance or the prevention of relapse; (3) *Comparator*: control group receiving either no treatment or treatment-as-usual (TAU); (4) *Outcome*: any measure indicative of AN psychopathology, e.g. Body Mass Index (BMI), Eating Disorder Examination (EDE) scores, percentage of relapse; (5) *Study design*: randomized controlled trial.

Case reports, narrative opinions or mere program descriptions were excluded as well as publications which did not undergo peer review.

### Risk of bias assessment

Following the Cochrane Handbook for Systematic Reviews of Interventions [24], we used the RoB 2 tool (https://doi.org/10.1136/bmj.l4898) to assess risk of bias for the primary outcomes of the included studies. Risk of bias assessment was performed by one rater (SCB). The RoB 2 tool assesses risk of bias in five domains (randomization process, deviations from intended interventions, missing outcome data, measurement of the outcome and selection of the reported result). The tool defines an algorithm to categorize risk of bias for each domain as well as the overall study in "low", "some concerns" and "high".

## Results

### Study selection

**Fig. 1:**
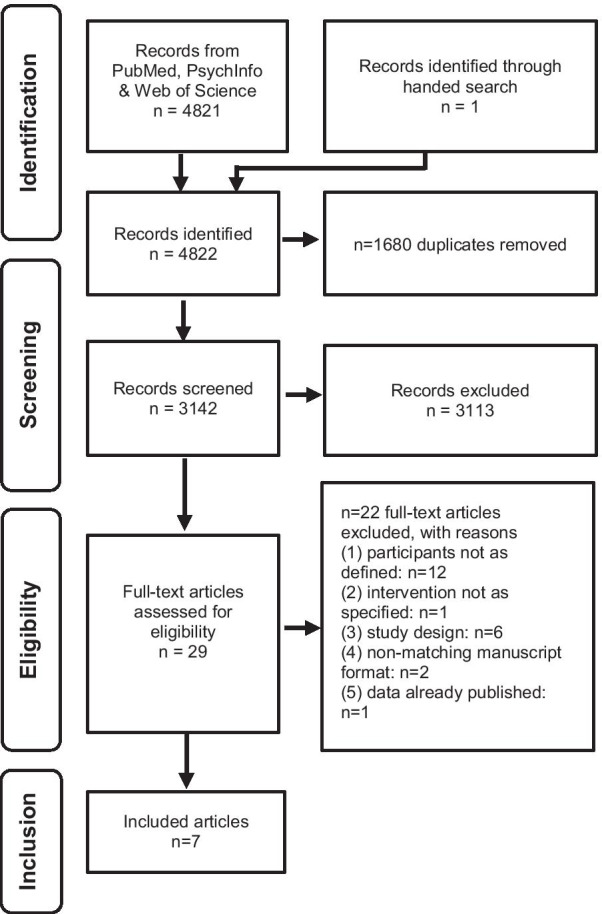
PRISMA Flowchart

Our systematic database search yielded 4822 results of published studies (see Fig. 1). After the screening process, 29 articles were included into the full-text assessment for eligibility. Of the 29 full-texts, 22 publications did not fulfill one or more of the inclusion criteria, leading to seven publications which were finally included into the in-depth analyses and summary. Agreement between raters was 87%. All included studies reported on aftercare interventions for adult patients with AN, while two trials reported data from mixed samples which also included adolescent patients [25, 26]. All included RCTs had a two-arm design. A total of 543 patients were included in these RCTs. Different interventions were used, including pharmacological agents, guided self-help approaches and psychotherapy. Table 1 gives an overview on core aspects and outcomes of each trial. As Table 2 shows, risk of bias within or across studies was moderate with the exception of one study with high risk of bias which was predominantly due to high dropout rates [27]. The calculated effect sizes for the trials ranged between -0.024 and 1.0 (Table 3). Additionally, our search on trial registration platforms yielded three ongoing registered RCTs [28–30].

**Table 1:** Overview on main characteristics and findings of RCTs investigating the efficacy of post-inpatient aftercare interventions for anorexia nervosa

Study	Aftercare intervention	Comparator	Adjunctive treatment	Duration	N	BMI at inclusion	Primary outcome	Definition of relapse	Main findings
Fichter et al. [28]	Internet-based guided self-help intervention (IGSH)	TAU	Outpatient or inpatient treatment and medication possible	9 months	258	IGSH: 17.8 ± 1.4 TAU: 17.7 ± 1.2	BMI	None provided	*Primary outcome* No significant group difference in weight gain after controlling for dosage of adjunctive inpatient treatment as the GSH group received more inpatient treatment as the TAU group *Secondary outcomes* Patients of the GSH had improved scores on some self-report dimensions related to ED cognitions and behaviors
Kaye et al. [25]	20 mg Fluoxetine/day (adjustment possible by a blinded physician)	Placebo	Optional adjunctive outpatient CBT	One year	35	89% average body weight	Prevention of relapse	Dropout from trial due to deteriorating clinical course (e.g. severe weight loss or severe ED symptoms), initiated by the patient, carer or physician	*Primary outcome* Significantly more patients receiving placebo had a relapse (dropped out) as compared to those receiving fluoxetine *Secondary outcomes* Patients who completed fluoxetine treatment over one year had higher weight, less ED symptoms, less obsessive thoughts, less depression and anxiety than the remaining group
Neumayr et al. [23]	Smartphone-based guided self-help intervention (SGSH)	TAU	SGSH is additional to TAU	8 weeks	40	SGSH: 19.1 ± 1.9 TAU: 18.6 ± 1.0	BMI self-reported ED symptoms	None provided	*Primary outcome* No significant group difference in weight gain or self-reported ED symptoms *Secondary outcomes* High levels of adherence and acceptance of SGSH
Parling et al. [29]	Acceptance and commitment therapy (ACT)	TAU	Optional additional daycare and other treatments; no additional psychotherapy for ACT patients	19 sessions	42	ACT: 17.5 ± 2.3 TAU: 18.1 ± 2.6	Good outcome defined as BMI ≥ 19 and EDE-Q global score ≤ 2.83	None provided	*Primary outcome* No significant group difference in good outcome *Secondary outcomes* Significant improvements in BMI and ED symptoms across time in both groups
Pike et al. [30]	Cognitive-behavior therapy (CBT)	Nutritional Counselling (NC)	Adjunctive pharmacotherapy possible	50 sessions over one year	33	Not reported BMI at admission: CBT: 16.0 ± 2.1 NC: 15.2 ± 1.5	Time to relapse	(a) BMI below 17.5 for more than 10 days (b) severe ED-related medical complications requiring inpatient care (c) exacerbation of non-ED psychopathology requiring other care	*Primary outcome* Patients in the CBT group had a significant longer relapse-free interval than patients receiving NC *Secondary outcomes* Patients in the CBT group showed a lower relapse rate and were more likely to meet criteria for good outcome
Sternheim et al. [26]	Internet-based guided self-help intervention based on MANTRA (iMANTRA) added to TAU	TAU	iMANTRA is additional to TAU	12 months	41	iMANTRA: 18.1 ± 2.2 TAU: 17.9 ± 1.4	Not defined due to feasibility focus	None provided	iMANTRA feasible and acceptable Effect sizes for BMI, ED pathology and general psychopathology at 6 months were small and tended to favor iMANTRA at 12 months assessment
Walsh et al. [24]	60 mg Fluoxetine/day (adjustment possible)	Placebo	Adjunctive outpatient CBT with specific focus on relapse prevention	One year	93	Fluoxetine: 20.2 ± 0.5 Placebo: 20.5 ± 0.5	Time to relapse	(a) BMI below 16.5 for 2 consecutive weeks (b) severe ED-related medical complications (c) imminent suicide risk (d) development of other severe psychiatric disorder	*Primary outcome* No difference in time to relapse between the fluoxetine and placebo group *Secondary outcomes* No difference in any secondary outcome between the fluoxetine and placebo group

ACT, Acceptance and Commitment Therapy; BMI, Body Mass Index; CBT, Cognitive Behavior Therapy; ED, eating disorder; EDE-Q, Eating Disorder Examination Questionnaire; IGSH, Internet-based Guided Self-help; iMANTRA, internet-based Maudsley Model of Anorexia Nervosa Treatment for Adults; NC, Nutritional Counselling; SGSH, Smartphone-based Guided Self-help; TAU, Treatment as usual

**Table 2:** Risk of bias assessment for included RCTs

Study	Randomization process	Deviations from intended intervention	Missing outcome data	Measurement of the outcome	Selection of reported results	Overall
Fichter et al. [28]	Some concerns	Some concerns	Low risk	Low risk	Low risk	Some concerns
Kaye et al. [25]	Low risk	Low risk	Low risk	Low risk	Some concerns	Some concerns
Neumayr et al. [23]	Some concerns	Some concerns	Low risk	Low risk	Some concerns	Some concerns
Parling et al. [29]	Low risk	Some concerns	High risk	High risk	Some concerns	High risk
Pike et al. [30]	Low risk	Some concerns	Low risk	Low risk	Low risk	Some concerns
Sternheim et al. [26]	Low risk	Low risk	Low risk	Some concerns	Low risk	Some concerns
Walsh et al. [24]	Low risk	Low risk	Low risk	Some concerns	Low risk	Some concerns

**Table 3:** Calculated effect sizes for the included RCTs

Study	n	*P* value main outcome	CI	Effect size
Fichter et al. [28]^a^	258	.076		.22
Kaye et al. [25]^a^	35	.006		.93
Neumayr et al. [23]^b^	40		− 0.90, 0.41	− .24
Parling et al. [29]^a^	42	.15	0.3–16.1	.64
Pike et al. [30]^a,c^	33	*p* < 0.004		1.00
Sternheim et al. [26]^b^	35		–0.28 to 1.09	0.40
Walsh et al. [24]^a^	39	.64		.20

^a^Calculation of effect size from *p*-value via t-value and sample size

^b^Calculation of effect size from CI via standard error and sample size

^c^*p*-value was set to 0.004

### RCTs reporting on pharmacological interventions

Two studies investigated the efficacy of fluoxetine versus placebo administration over one year following inpatient treatment for AN to prevent relapse [31, 32]. The rationale for testing pharmacological agents for relapse prevention in AN was that pharmacotherapy, which is largely ineffective in the treatment of acute AN [31–33], might unfold its effects in weight-restored patients [31], and that it might contribute to recovery by targeting common comorbidities such as depression [32].

In one of these trials [31], patients had to be weight restored at trial entry (minimum BMI of 19 kg/m^2^) and fluoxetine intake was adjunctive to individual cognitive-behavioral therapy (CBT) [34]. In the other trial, only patients suffering from the restrictive subtype of AN were included and an adjunctive outpatient psychotherapy was optional and taken up by some trial participants [32].

One of the trials determined *prevention of relapse* as primary outcome [32], and relapse was defined as dropout from the trial. Trial participation was terminated based on patient, physician or carer evaluation in case of a deteriorating clinical course of a patient, e.g. in terms of severe weight loss or severe ED symptom reoccurrence [32]. The second trial chose *time to relapse* as primary outcome [31], and relapse was defined based on several criteria reflecting severe deterioration of AN symptoms or comorbidities (see Table 1). In case of a relapse, trial participation was terminated [31].

The earlier and smaller trial reports limited intervention uptake with one third of eligible patients agreeing to participate, and a substantial dropout from the trial, especially in the placebo condition with only three out of 19 patients completing the study [32]. In contrast, 63% of participants of the fluoxetine arm remained in the study. This was a significant difference compared to the placebo condition, which was interpreted as reduced relapse rates in patients receiving fluoxetine. The second, larger trial reported that 20% of eligible patients had no interest in trial participation and that 57% of trial participants terminated the trial prematurely [31], with similar dropout rates in both arms. There was no significant group difference in time to relapse in this trial, indicating that fluoxetine had no benefit as a post-inpatient aftercare treatment for patients with AN [31].

### RCTs reporting on guided self-help interventions

Three trials investigated the efficacy of digital guided self-help interventions versus TAU following inpatient treatment for AN to prevent relapse [25, 26, 35]. The rationale for using digital treatment approaches in the aftercare for AN was to exploit the potential of these low-threshold interventions for patients living in a large catchment area and to make evidence-based treatments more available [35].

In one of these trials, patients were offered to participate in an internet-based program which involved nine modules of online self-help content together with monthly therapist-guided chats with other participants as well as email contact with therapists over nine months [35]. The self-help modules were based on principles of CBT and covered topics such as motivation and goal-setting, body acceptance, coping with ED symptoms and depression, emotion regulation, problem solving, social relationships and self-esteem [35]. In this trial, a minimum BMI increase during inpatient treatment was necessary for inclusion as well as “sufficient motivation” to take part in the study [35] which was defined by several criteria related to treatment history and compliance, including a prognostic assessment by the therapist. A later feasibility trial also offered a guided internet-based program, however, this was based on the MANTRA treatment concept, which is the Maudsley Model of Anorexia Nervosa Treatment for Adults [7, 26, 36, 37] and also involved regular email therapist contact. This intervention was offered over one year and in order to be included into this study, patients had to have a minimum weight gain of one BMI point during inpatient treatment [26]. Core aspects of the intervention which were specifically added to MANTRA for the aftercare intervention comprised a traffic light system of relapse risk, a nutritional plan designed for weight maintenance as well as module on anxiety-related processes [26]. In the other, more recent pilot RCT, patients were offered the use of an eight weeks long smartphone app *Recovery Record* as post-inpatient aftercare intervention [25]. This app includes interventions which are based on principles of CBT, dialectic-behavioral therapy (DBT) and motivational enhancement therapy (MET) [25], covering topics such as self-monitoring strategies, goal-setting, meal planning, coping strategies and guided meditations. Regular therapist contacts and feedback via the app were also included in this study [25].

One of these trials defined BMI as primary outcome [35], a further feasibility trial did not specify a primary outcome [26] and the second feasibility study primarily looked at BMI and self-reported ED symptoms as assessed by the Eating Disorder Examination Questionnaire (EDE-Q) [25]. None of these trials provided an explicit definition of relapse [25, 26, 35].

In the earlier larger study [35], 61% of eligible patients enrolled into the trial on internet-guided self-help, of those, 24.2% did not use the self-help program and 39.8% participants completed the full program. After controlling for adjunctive inpatient treatment, there was no significant group difference in weight gain between patients of the guided self-help group and those receiving TAU [35]. In the iMANTRA feasibility trial [26], 19.5% of inpatients screened for the study were found eligible and agreed to participate and 87.5% of those took up the iMANTRA intervention. Effect sizes for BMI, ED pathology and general psychopathology tended to favor iMANTRA at end of treatment, however, no significant differences were found [26]. In the later pilot study, 71.9% of eligible patients enrolled in the trial on smartphone-based guided self-help [25]. One patient terminated the intervention prematurely, all other participants showed regular daily use of the intervention [25]. At end of the aftercare intervention, there was no significant group difference in BMI or self-reported ED symptoms between those using the smartphone-based guided self-help adjunctive to TAU and those receiving TAU only [25]. The effect sizes in the feasibility trial at post-intervention were nonsignificant small to moderate favoring the intervention group regarding BMI (d =  − 0.24; 95% confidence interval [CI] [− 0.90, 0.41]) and ED symptoms (Eating Disorder Examination-Questionnaire global: d = 0.56; 95% CI [− 0.10, 1.22]) [25]. Effects between the groups were absent at 6-months follow-up [25].

### RCTs reporting on psychotherapy interventions

Two trials investigated the efficacy of psychotherapy interventions versus either TAU or nutritional counselling following inpatient treatment for AN to prevent relapse [27, 38]. One of the studies investigated a CBT intervention [38], while a second trial tested Acceptance and Commitment Therapy (ACT) as aftercare approach [27]. Differences in study design makes direct comparisons of the two trials difficult and each study is therefore reviewed separately.

#### Cognitive-behavior therapy (CBT)

In one trial, patients were randomly assigned to receive fifty sessions of either CBT or nutritional counselling as a control condition [38]. The CBT approach comprised topics including ED pathology, self-esteem and interpersonal functioning. Patients had to achieve a minimum weight gain up to 90% of ideal body weight maintained for at least two weeks during inpatient care in order to be eligible for the study [38]. *Time to relapse* was chosen as primary outcome [38], and relapse was defined similarly as in one of the later fluoxetine trials [31] based on several criteria reflecting severe deterioration of AN symptoms or comorbidities (see Table 1). In case of a relapse, trial participation was terminated [38]. Of those meeting inclusion criteria 76.7% took part in the study [38]. There was a high rate of treatment failure of 73% for the control group (a relapse rate of 53% plus a dropout rate of 20%), while the significantly lower rate of treatment failure in the CBT group was 22% (22% relapse, no dropout). There was a significant group difference in time to relapse, favoring CBT over the control condition [38].

#### Acceptance and commitment therapy (ACT)

Another trial investigated the efficacy of Acceptance and Commitment Therapy (ACT) as compared to TAU [27] for patients after intensive day-hospital treatment. Weight gain was not a primary focus of the day program, except for an optional final three weeks of the admission. Consequently, there was no eligibility criterion regarding minimum weight gain or BMI at randomization. The ACT therapy was a modified version of a treatment protocol developed for substance misuse (shortened from 48 to 19 sessions) and covered topics such as costs and benefits of the ED, emotional control and acceptance, experiential willingness, values and goals [27]. In both arms of the study patients were able to access additional clinical care if this was required. Primary focus of the analyses was change in BMI and in self-reported ED symptoms as assessed by the Eating Disorder Examination Questionnaire (EDE-Q) [25]; additionally, this trial compared proportions in both groups reaching a *good outcome,* defined as a BMI ≥ 19 and an EDE-Q global score ≤ 2.83. Roughly 84% of eligible patients consented to be enrolled into the trial, 41.7% of the ACT group completed less than 16/19 treatment sessions and significantly more (15.8%) dropped out of the TAU condition [27]. There was no significant group difference regarding good outcome at the end of the aftercare intervention [27].

### Ongoing trials

Our search for registered ongoing RCTs investigating the efficacy of aftercare interventions for AN [28–30] identified three studies which are currently in progress: The TRIANGLE study aims to strengthen self-management skills in a combined patient-carer approach [28]. A second study is based on the above outlined pilot RCT [25] testing the efficacy of a guided app-based self-help intervention as add-on to TAU in a larger sample [29]. The SUSTAIN trial conducted by our group investigates the efficacy of a novel post-inpatient psychotherapy which is predominantly delivered via videoconference [30].

### Operationalization of recovery processes

Most studies operationalized recovery/relapse through BMI, focusing either on time until the BMI fell below a cutoff, or on BMI at the end of the observation period. Further, all studies reported dropout rates as a marker of treatment adherence, although definitions of dropout varied considerably between studies.

## Discussion

The aim of this systematic review is to synthesize evidence from RCTs on the efficacy of treatments which aim to help patients with AN to maintaining benefits after inpatient or day-hospital treatment. We identified seven  RCTs, two of them with a pilot and feasibility focus. The interventions they tested can be classified into pharmacotherapy, guided self-help and psychotherapy approaches.

### Synthesis and discussion of results

The evidence from pharmacological efficacy trials is mixed, with one trial reporting superiority of fluoxetine vs. placebo in terms of reduced relapse rates [32] and a further, larger trial reporting no benefit of fluoxetine [31]. This heterogeneity could be due to methodological differences between the trials, concerning sample size, sample characteristics as well as definition and specificity of the primary outcome [31, 32]. It should also be considered that in both trials, adjunctive psychotherapy was included as part of the study protocol which also has an effect on the main outcome [31, 32]. Moreover, these studies suggest that post-inpatient pharmacotherapy is less acceptable to patients, as evidenced by low uptake and high dropout rates [31, 32]. Additionally, efficacy trials in this field of interventions are especially affected by ethical concerns about offering placebo treatments to severely ill patients [31, 32]. Pharmacotherapy plays a minor role in the treatment of AN as there is no evidence for its efficacy e.g. with respect to weight gain as a primary treatment goal [8, 13, 33]. In light of this, it is not surprising that there is no clear evidence for the efficacy of pharmacological agents in preventing relapse.

Guided self-help interventions have the potential to improve aftercare as they are more readily accessible and can be used more easily by patients who are ambivalent towards treatment. Moreover, they have the ability to bridge discontinuity of care, if disseminated via digital tools [39]. However, the efficacy of such digital guided self-help approaches as post-inpatient intervention for AN in terms of weight gain has not been demonstrated [25, 26, 35]. In one study, the self-help program was predominantly offered via homepage content, and the lack of superiority in this case might be partly due to a large proportion of patients not regularly engaging in the self-help program [35]. Those patients who fully completed the program did benefit [35], but the limited uptake might indicate reduced acceptability of this intervention, suggesting that this approach might not be intensive enough for a majority of patients. A later feasibility trial probing a guided internet-based self-help intervention in addition to TAU showed that this approach was well accepted and feasible, however, the trial was not powered to test efficacy [26]. A recent pilot study showed that contemporary mobile approaches like a smartphone application seem to meet many patients’ needs, in terms of ease of uptake, acceptance and satisfaction which was high [25]. However, the intervention had also a comparably short duration which tends to foster acceptability. The study did not demonstrate additional beneficial effects of using the app in comparison to the control group, but this needs to be seen in the context of a pilot RCT with the main focus on feasibility and acceptability [25]. The pilot data are encouraging regarding feasibility and acceptability and therefore, a larger appropriately powered confirmatory RCT probing the efficacy of the app is currently in progress [29]. Overall, the studies on internet-based guided self-help for patients with AN are adding important information to the literature in an emerging field, as a recent review outlines that studies investigating self-help have so far mainly focused on other ED diagnoses like bulimia nervosa (BN) and binge eating disorder (BED) [40]. For BN and BED, guided self-help interventions are superior to waitlist or delayed treatment regarding ED symptoms and abstinence rates [40], however, evidence is mixed and hard to interpret for comparisons with other (more intensive) treatments, partly also due to heterogeneous study designs [40]. Therefore, it will be important to investigate whether any form of self-help is a useful addition to current treatment options for AN, for instance, in terms of tailoring to patient needs with respect to content, speed and amount of guidance and feedback [41], but also regarding different levels of care and care pathways [12, 40–42].

The evidence on psychotherapy as aftercare strategy is mixed, too, with findings that a CBT intervention compared to nutritional counselling has the potential to delay relapse, while an ACT intervention did not result in better outcomes than TAU. Both trials had small sample sizes and variable dropout and treatment discontinuation [27, 38]. In the CBT study there was considerable drop out in the control condition, indicating that nutritional counselling was less accepted by patients [38], while in the ACT trial, engagement was actually higher in the TAU condition, although no difference was found in treatment outcome. In this latter trial, both study arms were able to access a range of additional treatment support [27], making it less likely to find differences.

To summarize, there is no clear evidence favoring a specific approach for post-inpatient aftercare in adult patients with AN. The effects of RCTs in this field are so far rather limited, partly due to various methodological challenges. Pharmacotherapy and low-threshold guided self-help suffer from limited uptake and high dropout [31, 32, 35]. Novel mobile guided self-help approaches seem more promising due to high patient satisfaction, but their efficacy has yet to be fully investigated [25, 29]. CBT might be beneficial in delaying relapse, but this is based on a single study with small sample size and high dropout in the control condition [38]. The CBT findings warrant replication in a larger well-powered trial. Recent reviews have emphasized that although considerable progress has been made in advancing evidence-based therapies for this patient group, there still is a long way to go [10, 21], and the present review shows that this also holds true for the more specific treatment stage of post-inpatient aftercare.

### Methodological aspects

As defined by the scope of the present review, all of the included studies had implemented a RCT design, which is a methodological strength, constituting the gold standard for efficacy research. With one exception [35], studies were conducted in small or modest samples, i.e. most have been insufficiently powered. This is also indicated by the calculated effect sizes which also underline the large heterogeneity of existing trials. Regarding sample size it should be taken into account that it is especially challenging to conduct large-scale RCTs in AN research, due to a lack of funding opportunities, low prevalence rates and ambivalence of patients [43]. Partly also related to patients’ ambivalence, most trials report comparably high dropout rates, especially from control conditions. Two major sources of heterogeneity between trials were choice of outcome as well as the sample characteristics. Some studies did not provide a definition of relapse, others did define relapse based on several outcome criteria and some studies primarily relied on BMI as primary outcome and indicator for relapse or recovery (see Table 1). This heterogeneity makes it difficult to compare trials, however, as outlined earlier, there is a general need for the field of ED to work towards a more unified definitions of relapse and recovery [20, 44]. Regarding sample characteristics, the BMI at inclusion into the aftercare intervention substantially varied between trials, and some studies defined a minimum weight gain, sufficient motivation or weight restoration as eligibility criteria [31, 35, 38]. This point again relates to the question how recovery or relapse are defined, and it is also tied to the situation that many adult patients with AN are not (weight) recovered or remitted when leaving inpatient or day-hospital treatment, posing the question to whom aftercare should be offered and tailored. A higher BMI at discharge from inpatient treatment is a positive prognostic predictor [45] and therefore is also likely to influence outcome of the aftercare intervention. At the same time, it could be argued that especially those patients who have not reached weight restoration are in need of ongoing support after discharge, as they have a higher relapse risk [6]. However, it might also be that this group of patients needs a specific form of aftercare. Finally, most trials allowed for or implemented adjunctive treatments as part of the trial (see Table 1). This was done for good reasons, especially regarding ethical and safety aspects in severely ill patients, nevertheless, it makes it hard to interpret the efficacy of a single novel aftercare intervention. For instance, pharmacotherapy was often combined with CBT, and therefore, the effects of psychotherapy might be to some extent responsible for a lack of group differences [31].

### Strengths and limitations of the present review

To the best of our knowledge, this is the first systematic review to summarize evidence on the efficacy of aftercare interventions specifically tailored at the treatment of patients with AN after inpatient or day-hospital treatment. We performed this work according to the PRISMA guideline for systematic reviews [23]. We have only included trials with an RCT design as this is the gold standard for efficacy research. We have used broad search terms, searched several large databases, performed an additional hand search and also included ongoing trials. Nevertheless, the number of included trials is small. Overall, the studies reviewed in this work are heterogeneous and disparate, and it is therefore important to take into account that this puts limitations on summarizing findings. Due to the heterogeneous sample characteristics and outcomes chosen, it was not possible to conduct a meta-analysis, however, we did calculate effect sizes and report the range. Further limitations comprise that the review was not pre-registered and that the quality rating was performed by one researcher.

### Future directions for research and treatment

Previous efficacy research demonstrates that there are several challenges in the development and implementation of effective aftercare for patients with AN: Patients with AN often experience ambivalence towards treatment [1], and also ‘treatment fatigue’. Especially after completion of intensive inpatient therapy, many patients might feel ambivalent towards continuing with another treatment. In this critical stage of transition, it is important to offer a treatment which meets patients’ needs, is not too low-threshold, and at the same time also fosters patients’ autonomy and supports them on their way to recovery. In light of this, the development and advancement of efficient aftercare interventions would benefit from stronger integration of the perspective of people affected by the disorder, either as a patient or carer. Their lived experience is invaluable for the development of tailored intervention modules, supporting those who are about to overcome the disorder. Two recent reviews synthesize findings from predominantly qualitative insights into personal views on the recovery process from an ED [20, 46], identifying fundamental aspects contributing to long-term recovery based on patient perspectives, and these contain surprisingly few domains which are directly tied to core ED symptoms or behaviors. Themes include social-emotional dimensions such as positive interpersonal relationships and personality-related aspects such as autonomy, developing an identity beyond the ED and self-acceptance [20, 46]. The ongoing trial by Cardi et al. [28] consequently implements this approach by investigating a novel combined patient-carer approach which partly relies on interventions which were developed by patients and carers. Integrating patients’ perspectives is also important towards the development of a consensus concept of recovery and relapse, as the use of heterogeneous outcomes in treatment trials is a further challenge in the field which makes comparability between interventions difficult.

Another issue which has yet to be investigated is that of interventions targeted to specific patient subgroups, for instance patients with the restrictive versus binge/purging subtype or those with comorbidities or longstanding illness. Overall, the variability between studies reviewed here could suggest that different people may benefit from different types of aftercare depending on the nature of previous treatment(s), stage of recovery, other support they have etc.

Beyond this, more research on mechanisms underlying the maintenance and progression of AN, but also recovery from this ED, will be important to inform novel treatment strategies, both for acute treatment as well as aftercare interventions [47]. Recently, novel innovative treatments which widely rely on insights from mechanism research have been probed for the treatment of AN, predominantly including non-invasive brain stimulation [21], and they might also unfold their effects in the stage of aftercare.

In order to extend current treatment options for adult patients with AN directly after inpatient or day-hospital treatment, our work group is currently conducting the SUSTAIN trial which is a multi-centre, randomized controlled confirmatory superiority trial investigating the efficacy of a novel post-inpatient psychotherapy as compared to optimized TAU [30]. The SUSTAIN aftercare intervention is based on the cognitive-interpersonal maintenance model of AN [7, 36, 37] and specifically tailored to achieve sustained recovery in AN following inpatient treatment. Patients randomized to the SUSTAIN aftercare intervention receive 20 treatment sessions over eight months. In order to ensure continuity of care for patients from a large catchment area, most treatment sessions take place via videoconference. Patients randomized to TAU-O receive routine outpatient psychotherapy. We will include 190 patients into this RCT who have reached a minimum BMI of 15 kg/m^2^ during acute treatment. Change in BMI between baseline and end of aftercare treatment (T2) adjusted for baseline BMI is the primary outcome. As part of the SUSTAIN trial, we have established a lived experience council whose members are patients with AN and carers. The council members contribute their perspectives throughout the whole trial period and have, for instance, also contributed to a revision of the treatment manual.

## Conclusion

Evidence on aftercare interventions for patients with AN is very limited, with a small number of randomized controlled intervention studies published. The field faces many challenges which generally affect intervention research in AN. Previous trials suggest that there lies potential in psychotherapy in terms of CBT-oriented aftercare interventions. Moreover, guided self-help approaches and the dissemination via digital dissemination strategies potentially have high acceptance and increase intervention uptake, however, their efficacy has yet to be demonstrated. A specific challenge is to increase uptake of aftercare interventions and to reduce dropout rates, calling for a better tailoring of interventions to patient needs and the integration of patient perspectives. Intensified research and care efforts are needed to address the problem of recurrent relapse after intensive inpatient treatment for AN and to eventually improve prognosis for this ED.

## Data Availability

Not applicable.
